# RustOnt: An Ontology to Explain Weather Favorable Conditions of the Coffee Rust

**DOI:** 10.3390/s22249598

**Published:** 2022-12-07

**Authors:** Carlos Suarez, David Griol, Cristhian Figueroa, Juan Carlos Corrales, David Camilo Corrales

**Affiliations:** 1 Departamento de Telemática, Facultad de Ingeniería Electrónica y Telecomunicaciones, Universidad del Cauca, Calle 5 No. 4-70, Popayán 190002, Colombia; 2 Department of Software Engineering, University of Granada, CITIC-UGR, Periodista Daniel Saucedo Aranda S/N, 18071 Granada, Spain; 3 Toulouse White Biotechnology (TWB), Institut National de Recherche Pour l’Agriculture, l’Alimentation et l’Environnement (INRAE), 135 Avenue de Rangueil, 31077 Toulouse, France

**Keywords:** coffee, ontologies, pest and disease management, rust, sensors, smart agriculture, software applications, weather

## Abstract

Crop disease management in smart agriculture involves applying and using new technologies to reduce the impact of diseases on the quality of products. Coffee rust is a disease that factors such as poor agronomic management activities and climate conditions may favor. Therefore, it is crucial to identify the relationships between these factors and this disease to learn how to face its consequences and build intelligent systems to provide appropriate management or help farmers and experts make decisions accordingly. Nevertheless, there are no studies in the literature that propose ontologies to model these factors and coffee rust. This paper presents a new ontology called *RustOnt* to help experts more accurately model data, expressions, and samples related to coffee rust and apply it whilst taking into account the geographical location where the ontology is adopted. Consequently, this ontology is crucial for coffee rust monitoring and management by means of smart agriculture systems. *RustOnt* was successfully evaluated considering quality criteria such as clarity, consistency, modularity, and competence against a set of initial requirements for which it was built.

## 1. Introduction

Smart or precision agriculture (PA) represents the application of information and communication technology (ICT) solutions in agriculture, such as the use of the Internet of Things (IoT), sensors and actuators, geopositioning systems, big data, unmanned aerial vehicles or drones, robots, etc. [1,2]. These technologies enable PA to present real potential for increased sustainability and agricultural productivity, improved economic returns based on the cost-effective use of inputs while reducing environmental impact, and resource preservation for the more efficient and accurate use of resources through decision support tools (DSTs) [3,4,5].

Smart agriculture also has clear environmental benefits, for example, through more efficient water use and optimizing the use of phytosanitary treatments for pest and disease management [6,7]. Coffee rust is caused by the fungus Hemileia vastatrix [8]. A coffee plant affected by this fungus presents the characteristics of the disease, such as yellow or orange powder on the underside of the leaves of the coffee plant in the form of spots or dots [9]. This disease causes the defoliation and drying of the branches and limited growth of the coffee plant, which consequently generates important losses in the production and quality of coffee [8]. The evolution of this disease can be favored by deficient agronomic management activities and some climatic conditions such as rain, humidity, temperature and sunlight. Therefore, the correct identification of these factors is crucial in the management of this disease.

A set of measures represent the status or value of meteorological variables in the field of coffee rust. The heterogeneity of these variables can cause inaccurate values and lead to misunderstandings in the interpretation of input data for models and systems developed to control coffee rust. In this regard, ontologies have been proposed to address data heterogeneity in different application domains [10,11]. Ontologies are a formal representation of knowledge and can be specified by concepts, taxonomies, functions, axioms, and instances [12,13]. Concepts are abstract or concrete representations of the real or fictional of virtually anything. Taxonomies are structures for organizing concepts. Relations are interactions between concepts. Axioms model statements that are asserted to be true in a domain. Finally, instances are elements of a domain attached to a specific concept [12,14].

Several research studies have documented the use of ontologies for agricultural control and monitoring for rice, coffee, and cocoa [15,16]; precision agriculture data [17]; mandarin crop life cycle [18]; coffee supply chain [19]; horticulture [20]; and smart livestock farming [21]. Only two of these studies are directly related to coffee cultivation and focus on modeling available pest and disease data. However, they lack a structure representing the climatic conditions favorable for coffee rust.

This paper proposes a new ontology, called *RustOnt*, for modeling favorable climatic conditions to prevent coffee rust. This ontology groups relevant concepts and instances of meteorological variables used by coffee rust control systems or models. In addition, *RustOnt* supports extensions to more concepts that can be detected later.

*RustOnt* allows experts to access a set of valid expressions, samples and values for each meteorological variable of interest in coffee rust, considering more precisely the geographical region (country) where the ontology can be adopted. This is possible because *RustOnt* has been constructed taking into account research studies on coffee rust models in several countries.

Early warning systems (EWSs) provide a set of articulated capacities, instruments and procedures for the purpose of monitoring, processing and systematizing information on foreseeable hazards in a specific area. The main objective is to reduce the loss of life and environmental damage, contributing to long-term sustainability [22]. The regional early warning system (SRAT) for rust and other important coffee diseases, developed by FONTAGRO for Central American and Caribbean countries, is an international cooperation platform for the development and co-financing of agricultural science and innovation projects [23]. The early warning and recommendation system (SART) to reduce rust growth was developed by the Coffee Institute of Costa Rica (ICAFE), which guarantees the quality and sustainability of coffee, promoting socially and environmentally responsible schemes [24]. *RustOnt* can contribute to the major objectives of these institutions, such as FONTAGRO and ICAFE, for Latin American and Caribbean countries through its integration into the SRAT and SART projects, helping to minimize the heterogeneous information used within the different modules that make up these EWSs.

The remainder of the paper is organized as follows. Section 2 describe the climatic variables considered after a detailed analysis of related work and the methodology selected to develop the *RustOnt* ontology. Section 3 and Section 4 respectively describe the *RustOnt* ontology, the process followed for its evaluation, and the main results obtained. Finally, Section 5 presents the main conclusions and future research lines.

## 2. Materials and Methods

This section presents the knowledge base needed to create our *RustOnt* ontology, the set of climatic variables considered after a detailed analysis of related work, and the methodology selected to develop the ontology.

### 2.1. Ontological Knowledge Base

An exhaustive study of the literature on the use of variables related to climatic conditions that can facilitate the detection of the incidence of rust in coffee has been carried out. Following the main steps of the systematic review methodology described in [25], the string of terms defined for the bibliographic search was (system OR model) AND (detection OR prediction) AND (coffee leaf rust OR coffee rust) AND (weather OR climatic) AND (incidence OR infection). Scopus, IEEE, Elsevier ACM and Springer data repositories were consulted, in addition to the reports and technical guides published by CENICAFE, ICAFE, and OIRSA. A total of 85 studies were obtained, 26 studies were selected, and 59 studies were discarded due the fact that they did not consider climatic variables and were only focused on concepts including the variety, density, main flowering, and initial infection level.

Table 1 shows the climatic variables considered in this study. The table details the climatic variables that favor coffee rust in various countries selected from the bibliographic study, the samples and values considered to develop the computational models, and the country where the research work was conducted. These primary variables (shadow, humidity, temperature, precipitation, and wind) have been considered to develop the knowledge base for the *RustOnt* ontology. The ontology focuses on the variability of these variables, scales, and specific values defined to measure the impact of rust coffee. Although the meteorological condition Altitude was considered in other studies, this work avoids including it in *RustOnt* because this condition does not include changes in samples or values for measurement over time. In the following subsection, we describe the methodology used to create *RustOnt*.

### 2.2. Overview of Methodologies to Build Ontologies

We have followed the work proposed in [48] to select the best methodology to develop the *RustOnt* ontology. Their work compares six methodologies to build ontologies (Uschold and Kings [49], Methontology [50], On-To-Knowledge [51], Noy, and McGuinness [52], Terminae [53], and Termontography [54]) based on the following criteria:

- C1: Intended audience that uses the ontology methodology.
- C2: Level of detail (scale 1–5). The methodology recommends the methods and techniques to use to perform the different activities.
- C3: Associated software application. The methodology recommends using a software application to build the ontology.
- C4: Conceptualization phase. The methodology organizes and structures the knowledge, independent from the knowledge representation paradigms and ontology languages. The representations must be comprehensible by domain experts and ontology developers through diagrams and tables.

Table 2 shows the previously described methodologies to build ontologies and the four criteria used for their comparison.

Based on the results of the comparison shown in Table 2, Methontology accomplishes the four criteria. This methodology is the most suitable for building ontologies due to its highly detailed instructions, good representation through charts and tables, and compatibility with popular ontology editors.

Methontology defines five phases or main tasks to be completed: (1) glossary of terms, (2) concept taxonomies, (3) ad-hoc binary relation diagrams, (4) concept dictionary, and (5) rules. The following section describes how *RustOnt* was created following the aforementioned phases.

## 3. The *RustOnt* Ontology

This section describes the process followed to develop the *RustOnt* ontology. This process has involved the selection of the set of terms included in the ontology, the definition of the taxonomy, ad hoc relationships, the concept dictionary, instances and class attributes, and rules.

### 3.1. Glossary of Terms

Table 3 shows the key terms included in *RustOnt*, as well as their descriptions, in natural language, and specific types (class, relation or attribute). Where a class represents a general term that involves similar characteristics for common individuals [55], a relation represents the association between individuals or classes [56], and the attributes are specific features associated to a class [57].

### 3.2. Concepts Taxonomies

A taxonomy formalizes the hierarchical relationships among concepts and specifies the term to be used to refer to each. It prescribes the structure and terminology of the ontology and provides a graphical view of concepts. The taxonomy selected for *RustOnt* employs a graph with rectangles representing classes, ovals as instances created from these classes, and lines denoting relationships. To develop the taxonomy, we have elaborated a glossary of terms verifying that there are not common instances among the concepts. In addition, we defined three general taxonomies for the following classes: Country, Mapping, and Weather_conditions.

**Figure 1:**
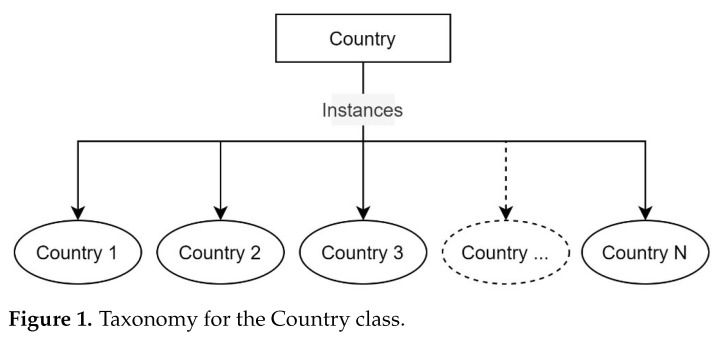
Taxonomy for the Country class.

The Country class presents a taxonomy that contains the countries where *RustOnt* can be applied, as Figure 1 shows. The Mapping class represents the set of possible transformations that can be performed with *RustOnt*. This class is used to transform textual values to their corresponding similar or matching values for an weather variable. The Sample class contains a type of transformation to obtain valid values or samples with units of time (e.g., day, monthly, seasonal, yearly, etc.), speed wind, temperature, humidity, or shadow conditions (e.g., percent, mm, Celsius, h/m, etc.) according to the computer models for the detection of coffee rust infections in each country. These values are instances of the weather variables supported by the *RustOnt* ontology.

**Figure 2:**
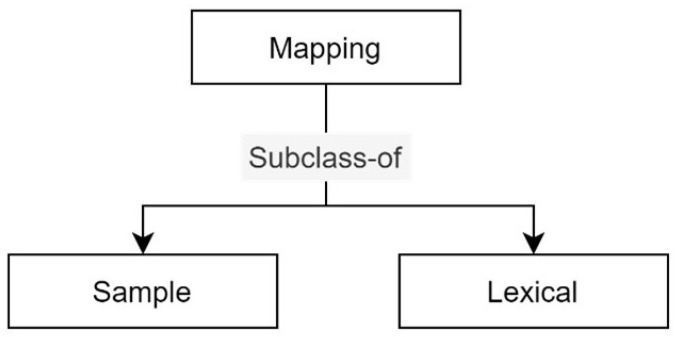
Taxonomy for the Mapping class.

The Lexical class denotes a type of transformation to obtain numerical values according to the synonym text or unit of time of the weather variables supported by *RustOnt*. The taxonomy to mapping is described in Figure 2, where Sample and Lexical have been defined as subclasses.

**Figure 3:**
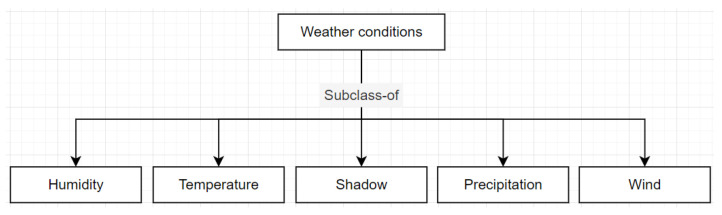
Taxonomy of the Weather conditions concept.

Figure 3 presents the Weather conditions class, in which there is an inheritance relationship with the Humidity, Temperature, Shadow, Precipitation, and Wind concepts.

### 3.3. Ad Hoc Binary Relation Diagrams

The ad hoc binary relation diagrams represent the relationships that connect a set of root concepts of the same or different taxonomies. Where each relation has a domain and range that should be assigned to the classes involved avoiding imprecise or over-specification. Concepts are represented with rectangles, and dotted lines denote their relations.

**Figure 4:**
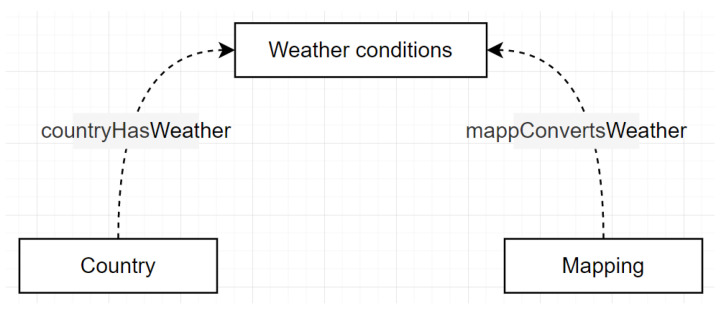
Ad hoc binary relation diagram defined for *RustOnt*.

The complete set of ad hoc relationships between the taxonomic concepts of the *RustOnt* ontology were described for this task. Figure 4 represents two existing binary relationships between the three main classes that were considered in the ontology.

- A country (1..1) has weather conditions (1..*): countryHasWeather
- A mapping (1..1) converts weather conditions (1..*): mappConvertsWeather

In addition, Table 4 presents the inverse relationships of the ad hoc binary relationships for the *RustOnt* ontology.

### 3.4. Concept Dictionary

The concept Dictionary is used to specify the characteristics for each of the classes defined in *RustOnt*, as shown in Table 5.

The Country class includes the name and three-digit international code features. The instances of this class correspond to countries that consider some variables belonging to the Weather Conditions class, which can be Temperature, Humidity, Shade, Precipitation, and Wind. The Mapping class is a type of class that denotes a transformation or mapping and is divided into two subclasses Lexical or Sample that applies the Converts relationship to transform the instances of the Weather Conditions class. The Weather Conditions class includes weather variables considered by *RustOnt*, e.g., the Humidity, Precipitation, Temperature, and Wind classes. These classes include different characteristics, such as conditions (greater than, less than), scale (average, minimum, maximum, etc.), minimum and maximum ranges, synonyms, time expressions (month, quarter, semester, etc.), and other units used by these variables. In addition, the Shadow class includes the following characteristics; synonyms, values, and expressions (equations and units).

### 3.5. Instance and Class Attributes

This task has been used to integrate the *RustOnt* instances selected from the literature survey described in Section 2.1. As described above, the main objective of this search was to obtain the most important climatic conditions used in computer models and technical reports to detect the incidence of coffee rust. Table 6 shows the instances of the *RustOnt* ontology and the bibliographic references that support their selection.

This task also describes the instance attributes created in *RustOnt*. Table 7 details the name of each attribute, corresponding classes, and data types (e.g., string, double, etc.).

### 3.6. Rules

Rules define a set of explicit rules to constrain the correct operation of *RustOnt*. This section describes the rules that are stored in the ontology, including the natural language description and the expression that formally describes each of them. To represent the set of rules, the semantic rules language (SWRL) was used, which allows expressing OWL concepts (classes, attributes, and instances) combined with RuleML to have a high-level syntax. Rule expressions follows the structure:

<antecedentsorconditions> (body) →<consequence> (head)

where antecedents or conditions are conjunctions of atoms $A_{1}$ ∧…∧ $A_{n}$ and functions $F_{1}\left(?A_{1},?A_{2}\right)$ ∧…∧ $F_{n}\left(?A_{n}\right)$, variables are represented with the character ‘?’ using the notation $?A_{n}$, and the consequence is a single atom.

A total of five rules have been defined for *RustOnt*. The rule (1) states that a Lexical class defines only instances that match a ’has’ relation applied:

(1) Lexical(?l)∧has(?h,?l)→sqwrl:select(?l,?h)

Rule (2) is used to list Lexical transformations corresponding to each Country instance:

(2) Country(?a)∧has(?h,?a)∧Lexical(?l)→sqwrl:select(?a,?h,?l)

Rule (3) lists only instances of Sample type that match a ’has’ relation applied:

(3) Sample(?s)∧has(?h,?s)→sqwrl:select(?s)

Rule (4) is only used to list instances of Sample type for each of the Country instances:

(4) Country(?a)∧has(?a,?h)∧Sample(?s)→sqwrl:select(?a,?h,?s)

Rule (5) restricts the converts and ’has’ relations on one same instance where they have been applied:

(5) converts(?c,?l)∧has(?h,?s)→swrlb:notEqual(?l,?s)

### 3.7. Ontology Editor

*RustOnt* has been modeled with the Protégé ontology editor. Protégé supports OWL 2, RDF, and XML schemes to create and edit ontologies. This software provides logical reasoners, such as HermiT and Pallet, to check for inconsistencies and support inference over the ontologies [61]. Protégé supports two types for ontology modeling: Protégé-Frames and Protégé-OWL [62]. This editor also provides a graphical user interface with tabs to model ontologies. The OntoGraf tab allows visualizing classes, instances, attributes and relationships in a graph. The entities tab allows managing data types, individuals and properties of annotations, data, classes, and objects. The individuals tab allows managing the instances of each class declared in the ontology. The DL query tab allows searching a classified ontology in a simple way using DL queries. The SPARQL tab supports SPARQL queries that provide a syntax for manipulating RDF graphs. Protégé also provides other tools that enable visualization and makes ontology maintenance easier.

The *RustOnt* ontology is publicly available at the following link: https://drive.google.com/file/d/1IoabvKSYBoVF1rM-P1lxTHh7X9GXbGk4/view?usp=sharing (accessed on 1 December 2022).

## 4. Evaluation

*RustOnt* has been evaluated to ensure the correct construction of its contents, definition, and implementation according to the requirements of the ontology and the competence issues that demonstrate the conformity between the actual model and the formal model [56]. In addition, we completed the evaluation of the competence and quality requirements that verify the correct behavior of the ontology with respect to the software environment, the documentation and reference framework created for its life cycle [63].

### 4.1. Ontology Competency

The competency of an ontological model denotes its ability to answer a set of questions [64]. This criterion is one of the most commonly used to evaluate ontologies [65,66,67] and to verify whether a model is complete with respect to a set of questions related to its competence [63].

The evaluation of this criterion is crucial to verify that a representation model is complete with respect to a set of competency questions [63]. The ontologist engineers and domain experts establishes the questions to be answered once the ontology has been implemented [63]. These questions are benchmarks to determine whether the model is sufficiently complete to represent the questions and solutions [64].

The competency evaluation of an ontology uses description logic (DL) axioms and SPARQL queries to model and answer an initial set of competency questions. We propose five competency questions to evaluate *RustOnt* (Q1–Q5). The evaluation runs on a laptop Asus S510U, ASUSTek cumputer Inc. Cali, Colombia with an Intel Core i7-8550U processor, 8 GB RAM, and a 64-bit Windows 10, @ 2019 Microsft corporation operating system.

**Figure 5:**
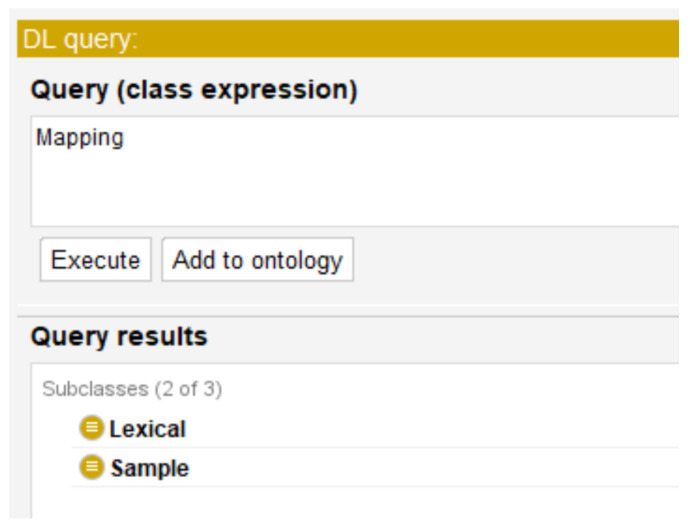
Execution of the DL mapping query using the Protégé editor and corresponding result, execution time was 25 ms.

**Figure 6:**
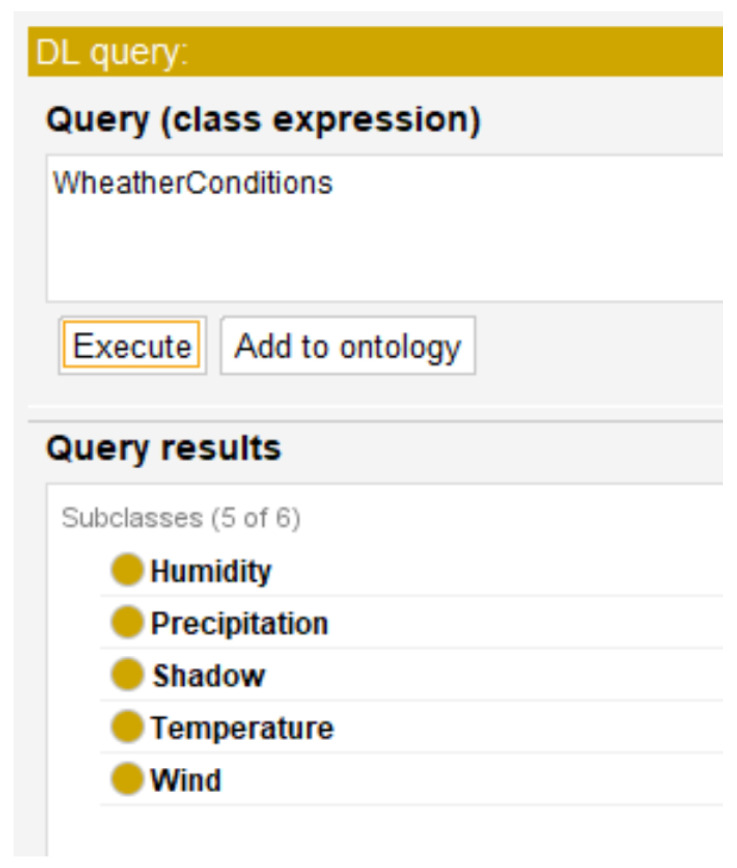
Weather conditions query using the Protégé editor and corresponding result, execution time was 40 ms.

**Figure 7:**
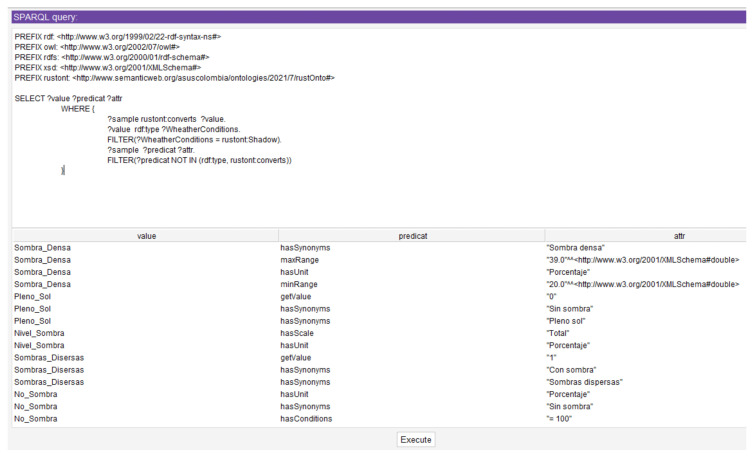
Query in SPARQL detailing the attributes of the supported values for the Shadow variable (Protégé 5’s Pellet reasoner, execution time was 194 ms).

**Figure 8:**
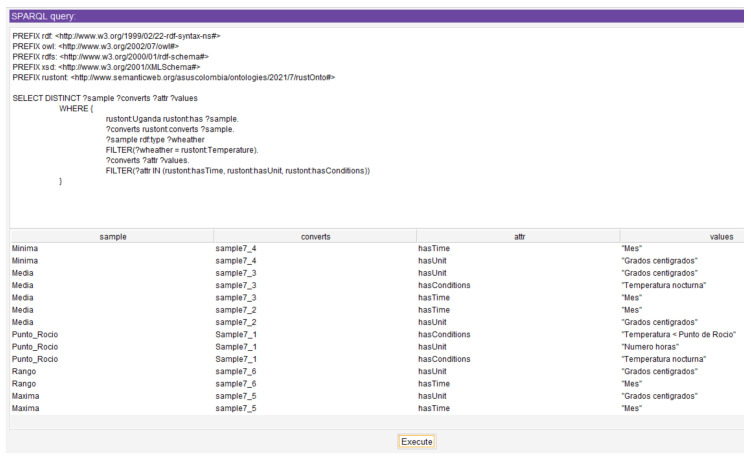
Query details the attributes of the valid samples for the query in Scenario 4 (Protégé 5’s Pellet reasoner, execution time was 264 ms).

Q1 What types of transformation or mapping are applicable to weather variables in computational models of coffee rust?
  The expected response of the ontology is based on the knowledge obtained through the bibliographical review of related work, identifying two ways of representing the values for the different variables [26,27,28,29,30,31,32,33,34,35,36,37,38,39,40,41,42,43,44,45,46,47,58,59,60].
  Lexical involves the use of different ranges to determine the value of a weather variable using a lexical expression or vocabulary [26,27,28,31,58,59,60]. Sample refers to the different samples or measurements that each work uses for each of the weather variables, in order to obtain the values with which their computational models have been developed [27,28,29,30,31,32,33,34,35,36,37,38,39,40,41,42,43,44,45,46,47].
  The following DL axiom (Equation (6)) allows describing the ontology’s answer to the competency question Q1:
  (6) $$\begin{array}{c} TBox=\{Mapping\equiv \exists hasName.xsd:string⊓\exists hasDescription.xsd:string,\\ Sample\subseteq Mapping,Lexical\subseteq Mapping\} \end{array}$$
  Figure 5 shows the sample and lexical transformation types supported by the designed ontology. These are subclasses of the Mapping class, which represents the types of transformation or mapping allowed by the ontology.
Q2 What are the weather variables taken into account in the computational models of coffee rust?
  From the bibliographic review, the weather variables Humidity, Precipitation, Shadow, Temperature, and Wind were identified as the most important ones for the development of computational models for the management of coffee rust [26,27,28,29,30,31,32,33,34,35,36,37,38,39,40,41,42,43,44,45,46,47,58,59,60].
  The following DL axiom (Equation (7)) allows describing the response of the ontology to the competency question Q2:
  (7) $$\begin{array}{c} TBox=\{Weather\_conditions\equiv \exists hasName.xsd:string⊓\exists hasDescription.xsd:string,\\ Humidity\subseteq Weather\_conditions,Temperature\subseteq Weather\_conditions,\\ Precipitation\subseteq Weather\_conditions,Shadow\subseteq Weather\_conditions,\\ Wind\subseteq Weather\_conditions\} \end{array}$$
  Using Protégé 5’s Pellet reasoner and executing a DL query (see Figure 6), the weather variables supported by the evaluated ontology are shown: Humidity, Precipitation, Shadow, Temperature, and Wind. There are subclasses of the Weather_conditions class, which groups all the different weather variables supported by the ontology.
  To solve the competency questions Q3, Q4, and Q5, a scenario is proposed for each question. The scenario approach is used to represent the queries in an ontology and modeled using the SPARQL language [63,67,68]. The solution to the proposed scenario indirectly answers each competency question.
  The following are the competency questions, the scenarios that are used as a tool to answer, the expected response against the scenario, the SPARQL query that represents the solution, and the result that is obtained from the ontology.
Q3 What are the meteorological variables considered by each country in the computational models of coffee rust?
  Scenario 1: In the South American country of Colombia, list the meteorological variables considered in the computational models developed for coffee rust. According to the bibliographical review, the meteorological variables considered by the computational models of coffee rust in Colombia are Temperature, Shadow, Humidity, Precipitation, and Wind, which are used in [29,30,33,37]
  The following SPARQL query corresponds to the solution for Scenario 1:
  **PREFIX** rdf: <http://www.w3.org/1999/02/22-rdf-syntax-ns#>
  **PREFIX** owl: <http://www.w3.org/2002/07/owl#>
  **PREFIX** rdfs: <http://www.w3.org/2000/01/rdf-schema#>
  **PREFIX** xsd: <http://www.w3.org/2001/XMLSchema#>
  **PREFIX** rustont: <http://www.semanticweb.org/asuscolombia/ontologies/2021/7/
    rustOnto#>
   
  **SELECT DISTINCT** ? object2
  **WHERE** {
rustont:Colombia rustont:has ? object1.
? object1 rdf:type ? object2.
**FILTER** (? object2 != owl: NamedIndividual)
}
  Table 8 presents the results obtained using *RustOnt* for the query corresponding to Scenario 1.
  Scenario 2: In the African region of Uganda, list the weather variables considered in the computational models developed for coffee rust.
  According to the bibliographical review, the weather variables considered by the computational models of coffee rust in Uganda are Temperature, Shadow, and Humidity [31,32].
  The following SPARQL query corresponds to the solution for Scenario 2:
  **PREFIX** rdf: <http://www.w3.org/1999/02/22-rdf-syntax-ns#>
  **PREFIX** owl: <http://www.w3.org/2002/07/owl#>
  **PREFIX** rdfs: <http://www.w3.org/2000/01/rdf-schema#>
  **PREFIX** xsd: <http://www.w3.org/2001/XMLSchema#>
  **PREFIX** rustont: <http://www.semanticweb.org/asuscolombia/ontologies/2021/7/
    rustOnto#>
   
  **SELECT DISTINCT** ? object2
  **WHERE** {
rustont:Uganda rustont:has ? object1.
? object1 rdf:type ? object2.
**FILTER** (? object2 != owl:NamedIndividual)
}
  Table 9 presents the results obtained for the query corresponding to Scenario 2 in *RustOnt*.
  Other scenarios can be proposed for countries such as Brazil, Republic of Costa Rica, Mexico, Belize, Guatemala, El Salvador, Honduras, Nicaragua, Panama, Dominican Republic, Ethiopia, Rwanda, and Papua New Guinea. These countries are also considered in *RustOnt* so that the proposed scenarios for these countries also find a response that satisfies competency question Q3.
Q4 Given a weather variable, what are the allowed values?
  Scenario 3: A user needs to know all the values supported by the ontology for the environment variable shade.
  According to the literature review, the allowed values for the environment variable shade are shade level, number of trees, no shade, thin shade, medium shade, dense shade, full sun, and scattered shadows [26,27,28,29,30,31,32]. The following SPARQL query corresponds to the solution of Scenario 3:
  **PREFIX** rdf: <http://www.w3.org/1999/02/22-rdf-syntax-ns#>
  **PREFIX** owl: <http://www.w3.org/2002/07/owl#>
  **PREFIX** rdfs: <http://www.w3.org/2000/01/rdf-schema#>
  **PREFIX** xsd: <http://www.w3.org/2001/XMLSchema#>
  **PREFIX** rustont: <http://www.semanticweb.org/asuscolombia/ontologies/2021/7/
    rustOnto#>
   
  **SELECT** ? value ? wheather
  **WHERE** {
? sample rustont:converts ? value.
? value rdf:type ? wheather.
**FILTER** (? wheather = rustont:Shadow)
}
  Table 10 shows the result of executing in *RustOnt* the query corresponding to Scenario 3.
  In addition, for the supported values of the Shadow variable, the attributes of each of the values can also be obtained through a SPARQL query as presented in Figure 7. For example, the attributes for the Fine_Shadow value that have properties such as hasUnit, maxRange, minRange that indicate attributes such as the supported unit which is “Percentage”, the minimum range “70” and the maximum range “99” supported for the Fine_Shadow value of the Shadow environment variable.
  Figure 7 shows how the attributes of each of the values supported by the Shadow variable can be obtained through a SPARQL query. For instance, the attributes for the *Fine_Shadow* value having properties such as hasUnit (e.g., “Percentage”), maxRange (e.g., 99), and minRange (e.g., 70).
  New scenarios can be constructed to query any of the weather variables (Temperature, Shadow, Precipitation, Wind, and Humidity). These scenarios can be evaluated using SPARQL queries that adequately satisfy the competency question Q4.
Q5 Given a value for an environment variable, what are the corresponding values it can take depending on the selected region?
  Scenario 4: A user in the African country of Uganda using *RustOnt* needs to know what values are allowed for the weather variable temperature.
  The values reported in the bibliographical review for the weather variable Temperature in Uganda are: daily mean, night mean, daily minimum, daily maximum, daily range, and number of hours when the temperature is below the dew point.
  The following query in SPARQL corresponds to the solution for Scenario 4:
  **PREFIX** rdf: <http://www.w3.org/1999/02/22-rdf-syntax-ns#>
  **PREFIX** owl: <http://www.w3.org/2002/07/owl#>
  **PREFIX** rdfs: <http://www.w3.org/2000/01/rdf-schema#>
  **PREFIX** xsd: <http://www.w3.org/2001/XMLSchema#>
  **PREFIX** rustont: <http://www.semanticweb.org/asuscolombia/ontologies/2021/7/
    rustOnto#>
   
  **SELECT**  **DISTINCT** ? sample ? environmental
  **WHERE** {
rustont:Uganda rustont:has ? sample.
? converts rustont:converts ? sample.
? sample rdf:type ? enviromental.
**FILTER** (?envirommental = rustont:Temperature)
}
  Table 11 shows the result obtained after executing the query corresponding to Scenario 4 in the *RustOnt* ontology.
  In addition, Figure 8 shows the SPARQL query with the attributes for the valid samples corresponding to Uganda. For instance, the example Dew_Point shows the hasUnit property with the value “Degrees Centigrade”, hasTime with the value “Night”, and hasConditions with value “Temperature < Dew Point”.
  As Scenario 4 was designed, it is possible to construct similar scenarios for other countries included in *RustOnt* by indicating the corresponding values for the weather variables (Temperature, Wind, Shade, Precipitation, and Humidity) on which SPARQL queries are desired. The results for similar scenarios show the details of the valid samples, as previously described for scenario 4. This allows satisfying the competence question Q5.
  As demonstrated for each of the initial competency questions, which have been solved through DL modeling or SPARQL queries and their respective answers, it is possible to conclude that *RustOnt* satisfies the competency requirement, as it provides a solution to a set of questions for which it developed.
  The results obtained demonstrate the effectiveness of each of the queries in satisfying the competency questions. Very fast response times ranging from 25 ms to 194 ms were obtained for the competency questions Q1, Q2, Q3, and Q4 and 264 ms for the competency question Q5, due to the number of instances supported by the ontology. As the number of instances increases, these times will surely change, however, this would be beyond the scope of the studies reviewed to date to create the instances for *RustOnt*.

### 4.2. Quality Requirement

The quality assessment of an ontology can be based on several principles for ontology design [69,70,71]. These principles include specific criteria and guidelines for designing and evaluating ontologies. Thanks to these principles, it is possible to determine the quality of an ontology based on the degree to which it meets the design criteria established from its design [63]. The criteria considered for the quality assessment of *RustOnt* are elaborated upon below.

#### 4.2.1. Clarity

According to [56,71], conceptual clarity can be defined as the capability of the ontology for the effective communication of the intended meaning of defined terms. For this purpose, formal axioms are defined that can be complete (necessary and sufficient conditions) or partial (necessary or sufficient conditions).

To satisfy this quality criterion, the ontology was first modeled by defining the formal axioms that were then implemented. Hierarchies were identified, data and object properties were designed and formally declared using a descriptive logic notation. Then, the main concepts of the ontology were defined. Lexical is a type of transformation that can be applied to some variables used by computational models for rust management in each country. It is defined with the pattern “Entity has name and description”. Lexical has the Precipitation, Wind, Temperature, and Shadow subclasses (Equation (8)).

(8) $$\begin{array}{c} TBox=\{Lexical\equiv \exists hasName.xsd:string⊓\exists hasDescription.xsd:string,\\ Precipitation\subseteq Lexical,Temperature\subseteq Lexical,Wind\subseteq Lexical,Shadow\subseteq Lexical\} \end{array}$$

Sample describes the different measures or values used by each country for the variables of the computational models for the management of coffee rust Sample is also defined as “Entity has name and description”. sample has the Precipitation, Temperature, Wind, Humidity, and Shadow subclasses (Equation (9)).

(9) $$\begin{array}{c} TBox=\{Sample\equiv \exists hasName.xsd:string⊓\exists hasDescription.xsd:string,\\ Precipitation\subseteq Lexical,Temperature\subseteq Lexical,Wind\subseteq Lexical,Shadow\subseteq Lexical\} \end{array}$$

*Precipitation* denotes the amount of rainfall that affects the coffee crop in a given period. It is defined as a subclass of Weather_conditions and has the name, description, synonyms, scale, maximum range, minimum range, time, and units properties (Equation (10)).

(10) $$\begin{array}{c} TBox=\{Precipitation\equiv \exists hasName.xsd:string\equiv \exists hasDescription.xsd:string⊓\exists \\ hasSynonyms.xsd:string⊓\exists hasScale.xsd:string⊓\exists \\ maxRange.xsd:float⊓\exists minRange.xsd:float⊓\exists \\ hasTime.xsd:string⊓\exists hasUnit.xsd:string,Precipitation\subseteq Weather\_conditions\} \end{array}$$

Temperature denotes the hot or cold conditions that affect the coffee crop in a given period. It is defined as a subclass of Weather_conditions and has the name, description, synonyms, scale, minimum range, maximum range, time, units, and expression properties as (Equation (11)).

(11) $$\begin{array}{c} TBox=\{Temperature\equiv \exists hasName.xsd:string⊓\exists hasDescription.xsd:string⊓\exists \\ hasSynonyms.xsd:string⊓\exists hasScale.xsd:string⊓\exists \\ maxRange.xsd:float⊓\exists minRange.xsd:float⊓\exists \\ hasTime.xsd:string⊓\exists hasUnit.xsd:string⊓\exists \\ hasExpresion.xsd:string,Temperature\subseteq Weather\_conditions\} \end{array}$$

Wind denotes the amount of wind that affects the coffee crop in a given period. It is defined as a subclass of Weather_conditions and has the name, description, synonyms, scale, minimum range, maximum range, time, and units properties (Equation (12)).

(12) $$\begin{array}{c} TBox=\{Wind\equiv \exists hasName.xsd:string⊓\exists hasDescription.xsd:string⊓\exists \\ hasSynonyms.xsd:string⊓\exists hasScale.xsd:string⊓\exists \\ maxRange.xsd:float⊓\exists minRange.xsd:float⊓\exists \\ hasTime.xsd:string⊓\exists hasUnit.xsd:string,Wind\subseteq Weather\_conditions\} \end{array}$$

Humidity denotes the water vapor content in the air during a given period of coffee cultivation. It is defined as a subclass of Weather_conditions and has the name, description, synonyms, scale, minimum range, maximum range, time, units, and expression properties (Equation (13)).

(13) $$\begin{array}{c} TBox=\{Humidity\equiv \exists hasName.xsd:string⊓\exists hasDescription.xsd:string⊓\exists \\ hasSynonyms.xsd:string⊓\exists hasScale.xsd:string⊓\exists \\ maxRange.xsd:float⊓\exists minRange.xsd:float⊓\exists \\ hasTime.xsd:string⊓\exists hasUnit.xsd:string⊓\exists \\ hasExpresion.xsd:string,Humidity\subseteq Weather\_conditions\} \end{array}$$

Shadow denotes the amount of shadow that the coffee crop has. It is defined as a subclass of Weather_conditions and has name, description, synonyms, scale, minimum range, maximum range, time, units, and expression properties (Equation (14)).

(14) $$\begin{array}{c} TBox=\{Shadow\equiv \exists hasName.xsd:string⊓\exists hasDescription.xsd:string⊓\exists \\ hasSynonyms.xsd:string⊓\exists hasScale.xsd:string⊓\exists \\ maxRange.xsd:float⊓\exists minRange.xsd:float⊓\exists \\ hasUnit.xsd:string⊓\exists hasExpresion.xsd:string,Shadow\subseteq Weather\_conditions\} \end{array}$$

**Figure 9:**
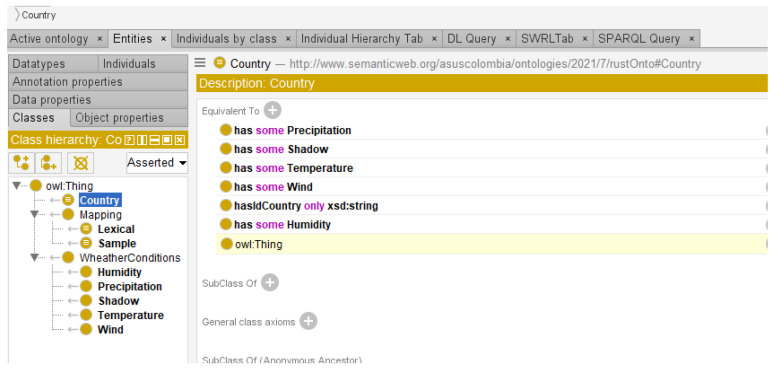
Complete definition for the country class (Protégé editor).

Complete definitions were made for the main classes Country, lexical, and sample. Figure 9 shows the concepts defined in the ontology and the complete definition for the concept Country.

#### 4.2.2. Coherence

**Figure 10:**
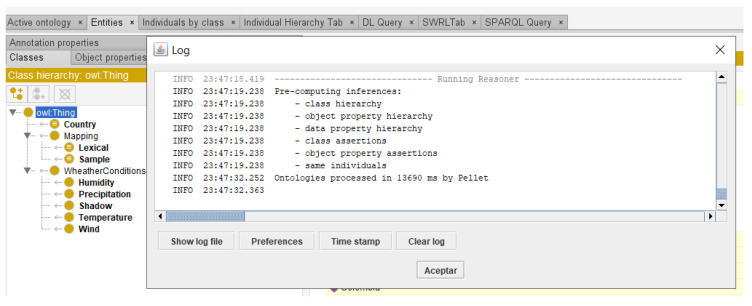
Use of Pellet reasoner to assess *RustOnt*’s coherence (the Protégé editor).

Coherence or consistency of an ontology denotes that inferred statements should be correct [58,63]. At the very least, the defining axioms should be logically consistent. In addition, the natural language documentation should be coherent with the formal statements. Figure 10 shows the use of the Protégé’s Pellet reasoner to assess the coherence of the *RustOnt* ontology. Using this tool, we verified that it is a consistent ontology model according to class hierarchy, object property hierarchy, data property hierarchy, and class assertions.

#### 4.2.3. Modularity

The modularity consists of decomposing an ontology into independent taxonomies [58,72]. This is a key factor for an ontology, since it allows its reusability and facilitates its maintenance and extension [72]. The modularity of *RustOnt* has been assessed by splitting the ontology model into two independent ontologies that can be applied in other domains.

**Figure 11:**
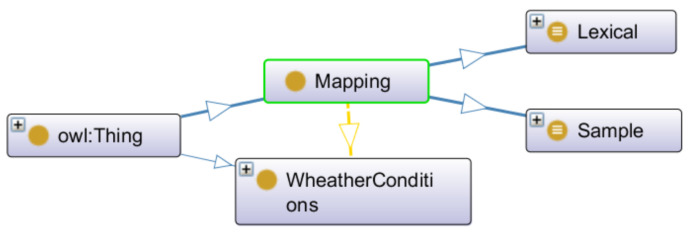
First ontology defined for the Mapping module (Protégé editor).

Figure 11 shows the first ontology defined for Mapping. It supports new types of transformation that can be additionally incorporated to the Weather_conditions instances.

**Figure 12:**
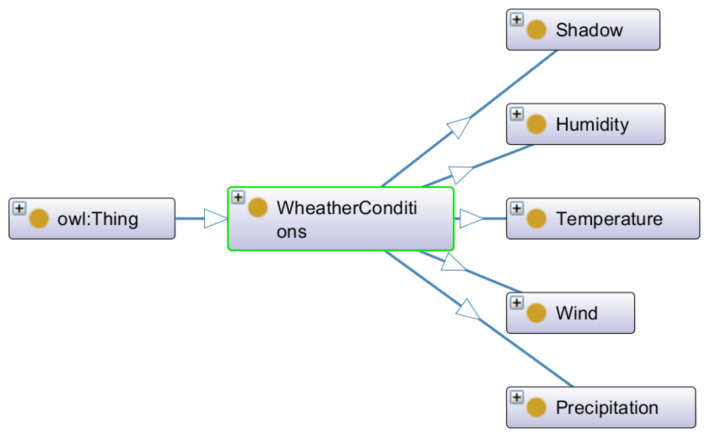
Second ontology defined for the Weather_conditions module (Protégé editor).

The second ontology has been defined for Weather_conditions (Figure 12). It supports adding new weather conditions corresponding to additional countries and adding new instances in mapping that can provide additional relationships.

Once the evaluation was completed, it was possible to verify that the competence criteria and quality requirements were successfully. This was performed using tools such as Protégé as well as DL queries through modeling scenarios with SPARQL queries and running the Pallet to Protégé reasoner inconsistency check, which was successful for the evaluated ontology. Therefore, it can be affirmed that the evaluated *RustOnt* ontology complies with the competence and quality requirements established in this work.

With the evaluation presented in this section, it has been possible to verify that the competence criteria and quality requirements were satisfactorily met by *RustOnt*. For this purpose, the tests described for each criterion have been completed using Protégé, DL queries, modeling scenarios with SPARQL queries, and inconsistency tests using the Pallet reasoner. From the results obtained, we can conclude that the *RustOnt* ontology meets the competence and quality requirements established for this work.

## 5. Conclusions and Future Work

Smart agriculture involves using information and communication technologies such as big data analytical techniques, data mining, cloud services, the Internet of Things, natural language processing (NLP), artificial intelligence (AI), and other strategies in agriculture and livestock farming. In this context, smart agriculture helps farmers optimize product quality, preserve natural resources and more effectively protect the environment.

Nevertheless, smart agriculture has created new challenges mainly related to using and exploiting the knowledge that these technologies have acquired and produced from crops and farmers. This knowledge needs to be collected, extracted, analyzed, and stored using mechanisms that allow farmers, experts, and machines to share common knowledge ground to address heterogeneous formats and data types.

Various methods have been developed for knowledge representation, including first-order logic, formal logic, semantic networks, frame-based systems, and ontologies. From these, ontologies are the most popular due to their ability to effectively analyze entities, usability, reusability, and maintainability.

In this paper, we proposed *RustOnt*, the first ontology with knowledge obtained from a literature review on the most critical weather variables in coffee cultivation and rust detection in Latin American and African countries. This ontology allows experts to build interoperable systems that exploit knowledge about the relationships between rust and critical weather variables to provide farmers with the information needed to address potential risks, reduce damage due to this disease and improve crop conditions, whilst keeping in mind that weather conditions that may favor its growth.

In addition, *RustOnt* allows experts to perform different transformations using the weather variables based on their specific values, units, and common expressions. The lexical transformation returns numerical values according to text values that farmers use to describe weather conditions (e.g., hot, cold, tempered, very cold, very hot, etc.) in different regions. The sample transformation provides the samples or valid values for each weather variable according to the selected region. These values and samples were obtained from related studies about computational models for coffee rust [26,27,28,31,59,60].

The evaluation of *RustOnt* was conducted on a question-based assessment focused on competency and quality requirements. The ontology successfully solved competency queries defined in DL. More complex queries required the definition of scenarios implemented using SPARQL queries. The quality assessment of *RustOnt* consisted of three criteria: clarity, consistency, and modularity. We defined formal axioms on the main classes to assess clarity, which allowed us to define the terms used and the hierarchy between them. The consistency was related to the clarity of each concept defined in the *RustOnt* ontology and its validation using the Pallet reasoner of the Protégé tool to infer knowledge about the elements of the ontology (classes, object property, data property and individuals) without contradictions. Furthermore, modularity was achieved by splitting *RustOnt* into two different modules (weather conditions and mapping), where each module is a representation ontology that can be used as an independent ontology for other domains.

Finally, *RustOnt* can help the process of data collection, integration, and knowledge exploitation in the field of coffee rust as an asset to create recommendation systems or predictive models that help farmers and experts in decision-making processes avoid adverse scenarios related to coffee rust.

The next step to improve *RustOnt* is to expand its information basis to include other coffee-producing countries that have not been considered due to the lack of previous studies. This objective can be achieved through initiatives such as the Regional Cooperative Program for the Technological Development and Modernization of Coffee (PROMECAFE) for Latin America and the Uganda Coffee Development Authority (UCDA).

We also want to integrate *RustOnt* as part of a case-based reasoning (CBR) system widely used in agriculture for crop management [3], traceability [73], yield estimation [74], and pest and disease protection [75,76,77]. *RustOnt* can improve the retrieval process of a CBR by making the input data less heterogeneous and thus more accurate, so that the search for the most similar case can be more precise according to the system requirements.

## Figures and Tables

**Table 1:** Climatic variables considered by country and their respective values, scales, and codification.

Climatic Variable	Values, Scales, and Codification	Country
Shadow	0 = full sun, 1 = Scattered shadows	El Salvador [26], Republic of Costa Rica [26,27,28]
	Shade percentage	Colombia [29,30]
	Not shade = 100%, fine shade = 99–70%, medium shade = 69–40%, dense shade = 39–20%	Uganda [31]
	Number of shade trees per hectare = shade trees/Ha	Uganda [32]
Humidity	average daily hours (>90%), average night hours (>90%), sum daily hours (>90%), sum night hours (>90%)	Colombia [30]
	Daily average relative humidity	Colombia [30,33]
	Number night hours (>95%)	Uganda [32]
	Minimum daily humidity (daily periods 14, 7, 4 and 3), average daily humidity (daily Periods 14, 7, 4 and 3)	Republic of Costa Rica [28]
	Amplitude relative humidity daily, leaf wetness time 6am–11am per day, moisture time in leaves (12am–6pm)/day	Republic of Costa Rica [34]
	Relative humidity under shade/hour, relative humidity full sun/hour	Republic of Costa Rica [27]
	Number of wet days/month	Mexico, Belize, Guatemala, El Salvador, Honduras, Nicaragua, Republic of Costa Rica, Panama, Dominican Republic [35]
	Yearly percentage	Ethiopia [36]
	(>80% monthly or quarterly), (<80% monthly or quarterly)	Colombia [37]
	Daily average relative humidity, number days relative humidity (>80%), number days relative humidity (>90%)	Brazil [38]
	Monthly average, number hours (>90% monthly) and (>80% monthly), number days with number hours (≥ 90% and > 6 h/month) and (≥80% and >6 h/month)	Brazil [39]
	Number of hours monthly (>90%) and (>80%), number days with number hours (≥90% and >6 h/month) and (≥80% and >6 h/month)	Brazil [40]
	Monthly or quarterly average, average number of hours relative humidity (>95% monthly or quarterly), average and maximum number night relative humidity (>95% monthly or quarterly)	Brazil [41]
	Average daytime hours (>95%), average and sum night hours (>95%), sum daylight hours (>95%)	Brazil [42]
	Number of daytime hours (>95%), number of night hours (>95%)	Brazil [43,44]
	Average relative humidity last 2 months, relative humidity (>90% last 2 months)	Colombia [29]
Temperature	Average during day (>90%), average overnight (>90%)	Colombia [30]
	Daily maximum, daily minimum, daily average	Colombia [30,33]
	Thermal amplitude	Mexico, Belize, Guatemala, El Salvador, Honduras, Nicaragua, Republic of Costa Rica, Panama, Dominican Republic [35]
	Daily maximum, minimum and average (daily periods of 14, 7, 4 or 3)	Republic of Costa Rica [28]
	Yearly average	Ethiopia [36]
	Variation of the temperature last month	Colombia [29]
	Daily average, night average, daily minimum, daily maximum, daily range, number of hours (temperature is below dew point at night)	Uganda [32]
	Daily air temperature: minimum, maximum and thermal amplitude, daily leaf temperature: minimum, maximum and thermal amplitude	Republic of Costa Rica [34]
	Thermal amplitude: (semi-annual/quarterly periods) = Temp. Max − Temp. Min, small (temperatures < 12), large (temperature > 12)	Colombia [37]
	Monthly: minimum-maximum; annual: minimum average-maximum average	Rwanda [45]
	Accumulated monthly (maximum, minimum, and average), seasonal period (accumulated minimum, accumulated maximum, average, monthly variation, seasonal variation and climatological variation)	Guatemala [46]
	Daily minimum, daily maximum	Papua New Guinea [47]
	Average daily maximum temperature, average daily minimum temperature	Brazil [38]
	Monthly average, monthly minimum, monthly maximum, average temperature with relative humidity (>80%), average temperature with relative humidity (>90%)	Brazil [39]
	Monthly average, monthly minimum, monthly maximum	Brazil [40]
	Average, maximum temperature, average minimum and minimum temperatures (monthly, quarterly, seasons), average temperature with hours of relative humidity (>95% monthly, quarterly, seasons), maximum temperature hours with relative humidity (>95% monthly, quarterly, seasons)	Brazil [41]
	Average daily temperature with relative humidity (>95%), average temperature of daily maximums, average temperature of daily maximums (incubation period), average daily temperature, average daily temperature (incubation period), average temperature of daily minimums, average temperature of daily minimums (incubation period)	Brazil [42]
	Average daytime temperature relative humidity (>95%), average night temperature relative humidity (>95%)	Brazil [43,44]
Precipitation	Number of days (precipitation > 1mm, daily periods 14, 7, 4 or 3), daily precipitation (daily periods 14, 7, 4 or 3)	Republic of Costa Rica [28]
	Number of rainy days last month, accumulated rainfall last 2 months, accumulated night rainfall last month	Colombia [29]
	Average daily rainfall, accumulated average daily rainfall	Colombia [30]
	Annual precipitation	Ethiopia [36]
	Monthly precipitation	Rwanda [45]
	Total daily rainfall, duration of daily precipitation	Republic of Costa Rica [34]
	Rainfall(mm) every hour daily, number of daily hours without precipitation (<0.1 mm), number of daily hours of precipitation (>0.1 mm)	Republic of Costa Rica [27]
	Accumulated monthly, accumulated seasonal	Guatemala [46]
	Total daily (mm), number of days of precipitation (≥1 mm and <9 mm), number of days of precipitation (>10 mm)	Brazil [38]
	Monthly total, number of days with precipitation (>1 mm/month), number of days with precipitation (≥20 mm/month)	Brazil [39]
	Number of days with precipitation (≥1 mm/month), number of days with precipitation (≥20 mm/month)	Brazil [39,40]
	Average accumulated precipitation (monthly, quarterly, seasons), accumulated precipitation (monthly, quarterly, seasons)	Brazil [41]
	Number of days with precipitation (≥1 mm), average daily, average of maximum, accumulated daily	Brazil [42]
Wind	Average daily speed (m/s)	Colombia [30]
	Every hour (m/s)	Republic of Costa Rica [27]
	Average daily speed Hm/h, sum of daily average speed Hm/h	Brazil [42]

**Table 2:** Comparison of methodologies to build ontologies; source: [48].

Methodology	C1	C2	C3	C4
Uschold and Kings [49]	Ontology developers	3	No	No
Methontology [50]	Ontology engineers and researchers	5	WebODE and Protégé	Yes
On-To-Knowledge [51]	Ontology developers	4	OntoStudio	Yes
Noy and McGuinness [52]	Ontology developers	5	Protégé	No
Terminae [53]	Knowledge engineers and terminologists	4	Terminae	Yes
Termontography [54]	Ontology builders, terminographers, and lexicographers	3	Termontography Workbench	No

**Table 3:** Description and type (class, relation, or attribute) of the *RustOnt* terms.

Name	Description	Type
Mapping	Types of transformation that the ontology supports	Class
Lexical	Subclass of Mapping used to assign numerical scales to variables expressed as text	Class
Sample	Subclass of Mapping used to show valid values for weather variables depending on a specific country	Class
Country	Countries where the coffee rust has been studied	Class
Weather conditions	Samples of weather variables that affect coffee rust	Class
Humidity, precipitation, shadow, temperature, wind	Specific class in the set of weather conditions	Class
Value	This field can contain a specify type or unit (e.g., %, °C)	Attribute
Conditions	Restrictions of type greater than or less than, equal and if for the values of weather variables	Attribute
Expression	Mathematical description for the values of weather variables	Attribute
IdCountry	Alphabetic code associated with an instance of the Country class	Attribute
Range	Limits for the values of weather variables	Attribute
Synonyms	Lexical expressions for a value of an weather variable	Attribute
Time	Timestamp of a value of an weather variable	Attribute
Unit	Units used to specify the values of an weather variable	Attribute
Converts	Transformations used for weather variables	Relation
Has	Indicates the existence of weather variables for a country	Relation

**Table 4:** Reverse relations of ad hoc binary relations from RustOnt.

Relation Name	Inverse Relation
Country has weather conditions	Weather conditions of a country
Mapping converts weather conditions	Weather conditions are used to apply a mapping

**Table 5:** Concept dictionary for the Country, Mapping, Sample, Lexical, Weather conditions, Humidity, Temperature, Shadow, Precipitation, and Wind variables.

Class Name	Class Attributes
Country	Name and 3-digit international code that represents the country.
Mapping	Class type transformation can be lexical or sample.
Sample, lexical	A subclass of mapping that contains the converts relation.
Weather conditions	A class type for weather conditions.
Humidity, temperature, precipitation, wind	Attributes: scale, sample, conditions, minimum and maximum ranges, synonyms, units of time and units of the value of the variable.
Shadow	Attributes: synonyms, the correspondence value, expressions, and the units of the value of the variable.

**Table 6:** Instances and class attributes for the Country, Sample, Lexical, Humidity, Temperature, Shadow, Precipitation, and Wind variables.

Class Name	Instances
Country	20 instances [26,27,28,29,30,31,32,33,34,35,36,37,38,39,40,41,42,43,44,45,46,47]
Sample	142 instances [27,28,29,30,31,32,33,34,35,36,37,38,39,40,41,42,43,44,45,46,47]
Lexical	32 instances [26,27,28,31,58,59,60]
Humidity	42 instances [27,28,29,30,32,33,34,35,36,37,38,39,40,41,42,43,44]
Temperature	67 instances [28,29,30,32,33,34,35,36,37,38,39,40,41,42,43,44,45,46,47]
Shadow	8 instances [26,27,28,29,30,31,32]
Precipitation	37 instances [27,28,29,30,33,34,36,38,39,40,41,42,45,46,59]
*Wind*	17 instances [27,30,42,60]

**Table 7:** Attributes for the hasSynonyms, hasScale, maxRange, minRange, hasUnit, hasExpresion instances.

Instance Attributes	Class Name	Type
hasIdCountry	Country	xsd:string
getValue	Lexical	xsd:string
hasExpresion	Shadow, Temperature, Humidity	xsd:string
hasTime	Precipitation, Temperature, Wind, Humidity	xsd:string
hasSynonyms, hasScale, hasConditions, hasUnit	Precipitation, Shadow, Temperature, Wind, Humidity	xsd:string
hasRange (maxRange, minRange)	Precipitation, Shadow, Temperature, Wind, Humidity	xsd:double

**Table 8:** Query result for scenario 1, using Protégé 5’s Pellet reasoner, execution time was 33 ms.

Object2
Temperature
Shadow
Humidity
Precipitation
Wind

**Table 9:** Query result for scenario 2, using Protégé 5’s Pellet reasoner, execution time was 38 ms.

Object2
Temperature
Shadow
Humidity

**Table 10:** Query result for Scenario 3 using Protégé., Weather indicates the name of the class and Value denotes the allowed values for the class, execution time was 74 ms.

Weather	Value
Shadow	Number trees
Shadow	Dense shadow
Shadow	Full sun
Shadow	Shadow level
Shadow	Sparse shadows
Shadow	No shadow
Shadow	Media shadow
Shadow	Fine shadow

**Table 11:** Result of the query for Scenario 4. Each sample line shows the values accepted for the weather variable Temperature (Protégé 5’s Pellet reasoner, execution time was 110 ms).

Sample	Weather
Minimum	Temperature
Mean	Temperature
Dew_point	Temperature
Range	Temperature
Maximum	Temperature

## Data Availability

Not applicable.

## Abbreviations

The following abbreviations are used in this manuscript:

AI	Artificial Intelligence
CENICAFE	National Coffee Research Center of Colombia
DSTs	Decision Support Tools
EWSs	Early Warning Systems
FONTAGRO	Regional Agricultural Technology Fund
ICAFE	Costa Rica Coffee Institute
ICT	Information and Communication Technology
IoT	Internet of Things
OIRSA	International Regional Organization for Animal and Plant Health
NLP	Natural Language Processing
PA	Smart or Precision Agriculture
SPARQL	SPARQL Protocol and RDF Query Language
SRAT	Regional Early Warning System
